# Interventional Procedures for Vertebral Diseases: Spinal Tumor Ablation, Vertebral Augmentation, and Basivertebral Nerve Ablation—A Scoping Review

**DOI:** 10.3390/healthcare9111554

**Published:** 2021-11-15

**Authors:** Vincius Tieppo Francio, Benjamin Gill, Adam Rupp, Andrew Sack, Dawood Sayed

**Affiliations:** 1 Department of Rehabilitation Medicine, The University of Kansas Medical Center (KUMC), Kansas City, KS 66160, USA; arupp3@kumc.edu; 2 Department of Physical Medicine and Rehabilitation, The University of Missouri, Columbia, MO 65212, USA; benjamingill@health.missouri.edu; 3 Department of Anesthesiology, The University of Kansas Medical Center (KUMC), Kansas City, KS 66160, USA; asack@kumc.edu (A.S.); dsayed@kumc.edu (D.S.)

**Keywords:** low back pain, spinal neoplasm, vertebral fracture, vertebrogenic pain, spinal tumor ablation, basivertebral nerve ablation, vertebral augmentation, kyphoplasty, vertebroplasty

## Abstract

Low back pain is consistently documented as the most expensive and leading cause of disability. The majority of cases have non-specific etiologies. However, a subset of vertebral diseases has well-documented pain generators, including vertebral body tumors, vertebral body fractures, and vertebral endplate injury. Over the past two decades, specific interventional procedures targeting these anatomical pain generators have been widely studied, including spinal tumor ablation, vertebral augmentation, and basivertebral nerve ablation. This scoping review summarizes safety and clinical efficacy and discusses the impact on healthcare utilization of these interventions. Vertebral-related diseases remain a top concern with regard to prevalence and amount of health care spending worldwide. Our study shows that for a subset of disorders related to the vertebrae, spinal tumor ablation, vertebral augmentation, and basivertebral nerve ablation are safe and clinically effective interventions to decrease pain, improve function and quality of life, and potentially reduce mortality, improve survival, and overall offer cost-saving opportunities.

## 1. Introduction

As the most expensive condition, over USD 100 billion dollars per year, and the top cause of disability worldwide, prevalent in up to 70–80% of adults, low back pain (LBP) is a prime target for effective treatments [1,2,3,4]. LBP has a non-specific etiology in the majority of individuals (up to 80–90%) [5,6,7]. The complexity of treatments for LBP can be traced to the multiple anatomical structures that may contribute to symptoms, including intervertebral discs, ligaments, muscles, spinal nerve roots, and lumbosacral zygapophyseal facet and sacroiliac joints [5,7,8]. Recognition of specific etiologies allows for precise interventions and optimization of clinical outcomes. There are a variety of interventional pain procedures to target these anatomical pain generators, each with varying levels of efficacy given the often vague diagnosis.

Disorders specific to the vertebrae, on the other hand, have fairly distinctive anatomical etiologies, such as vertebral body tumors, vertebral body fractures, or vertebral endplate disruption or inflammation. These can be addressed by interventional pain procedures, such as vertebral body spinal tumor ablation (STA), vertebral augmentation (VA), and basivertebral nerve ablation (BVNA). Therefore, our review aims to describe the pathoanatomical and diagnostic findings of these etiologies and the safety and clinical effectiveness of these interventions in the management of highly prevalent and costly vertebral disorders.

## 2. Methods

**Figure 1:**
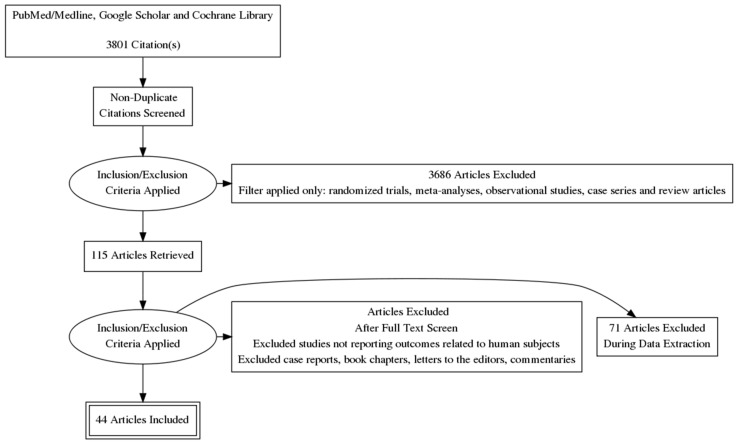
PRISMA-ScR flow chart methodology with identification, screening, eligibility and inclusion and exclusion process. Adapted from: Tricco, AC, Lillie, E, Zarin, W, O’Brien, KK, Colquhoun, H, Levac, D, Moher, D, Peters, MD, Horsley, T, Weeks, L, Hempel, S, et al. PRISMA extension for scoping reviews (PRISMA-ScR): checklist and explanation. Ann Intern Med. 2018,169(7):467-473 [9].

This study is a scoping review aimed at appraising vertebral disorders, pathoanatomical considerations, diagnostic findings, clinical efficacy, and safety of interventional pain management procedures. Data sources included PubMed, MEDLINE, Google Scholar, and Cochrane Library indexed manuscripts. The literature search was conducted between May 2021 and August 2021 using the following keywords: vertebral body spinal tumor ablation, vertebral augmentation, and basivertebral nerve ablation. Inclusion criteria were human studies in the English language, such as randomized trials, meta-analyses, observational studies, case series, and review articles. All records identified in the search were independently appraised by two reviewers in a standardized, unblinded fashion, using the same strategy to ensure proper cross-checking of the results with the preferred reporting items for systematic reviews and meta-analyses extension for scoping reviews (PRISMA-ScR) methodology (Figure 1). The PRISMA-ScR flow diagram and process were used to reduce selection bias and standardize inclusion and exclusion criteria [9]. Any disagreement regarding accepting studies was resolved by a discussion until consensus was reached. Single case reports, book chapters, commentaries, and letters to the editor were excluded. Data extracted from the included studies consisted of the date of the study, authors, journal, study design, core components, and primary outcomes. For all studies, data synthesis and analysis were performed with assessments of risk of bias, quality, and outcome measurements by two authors independently, thereafter reviewed by all authors.

## 3. Results

Our search found 3801 studies with the selected keywords. Of these, 115 studies were filtered based on our inclusion criteria and reviewed. Seventy-one studies were excluded based on predetermined criteria of original studies related to human subjects related to STA, VA, and BVNA and using the PRISMA-ScR protocol. Forty-four studies were included in our data analysis. A summary of clinically and statistically significant findings from landmark studies and level I and level II studies were compiled, qualitatively analyzed, and reported on Table 1, Table 2 and Table 3, along with relevant comments comprehensively outlining the details of each study, statistical findings, sample size, treatment groups, and adverse events.

## 4. Discussion

Vertebral disorders are associated with significant socioeconomic and medical sequelae due to high prevalence and heavy burden in health care cost utilization [54]. Appropriate management of LBP is interdisciplinary in nature, focusing on rehabilitation and interventional pain management procedures guided by the specific anatomic pain generator. Although the majority of LBP is nonspecific, subset etiologies of vertebral pain may be spinal or vertebral tumors, vertebral fractures, and vertebrogenic pain from endplate disruption, which can be targeted by specific interventions such as STA, VA, and BVNA, respectively. The studies identified in this scoping review represent the current evidence regarding these interventions in vertebral pathologies. This evidence may provide guidance and support clinical and policy decision-making in the treatment of these very prevalent, debilitating and highly costly vertebral-related disorders.

### 4.1. Vertebral Body Tumors

**Figure 2:**
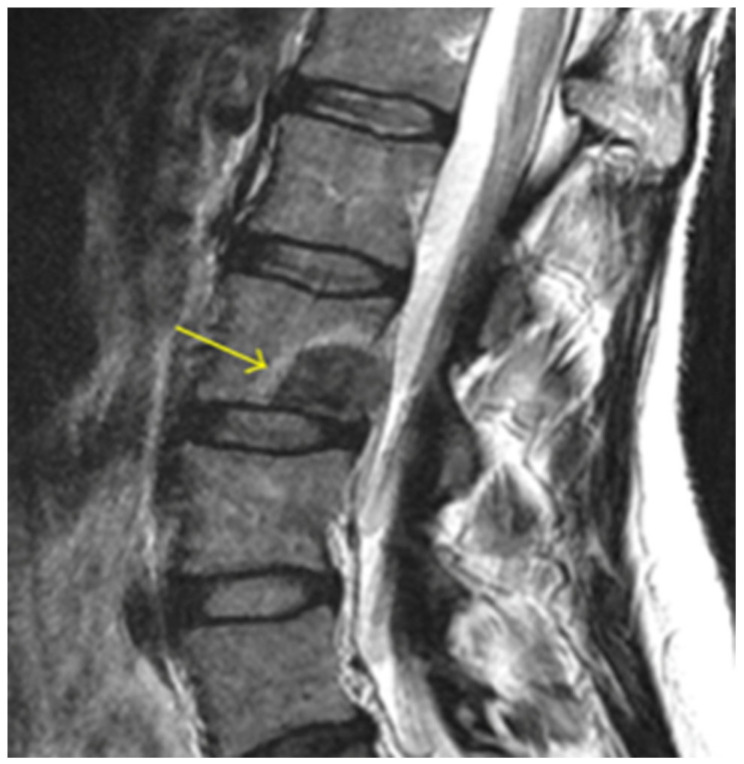
Lumbar spine magnetic resonance image (MRI) with an arrow pointing at the posterior–inferior edge of the vertebral body, highlighting a vertebral body tumor.

Vertebral tumors are benign or malignant growths that involve the vertebral body of the spinal column (Figure 2). Nearly all malignancies are the result of metastasis (97%) rather than primary solid vertebral body tumors. The spine is affected in 30–70% of metastatic diseases with the vertebral bodies, especially throughout the thoracic and lumbar spine, cited as the third most common site for osseous metastasis [55,56]. This association is largely a function of the rich vascular and lymphatic connections to common sites of cancerous tissue throughout the thorax and pelvis [57]. The spine is involved in 65–75% of breast and prostate malignancies, 30–65% of lung cancers, more than 40% of metastatic thyroid cancers, and about 30% of renal cell carcinomas [58,59,60,61]. Although much less common, primary spinal tumors include multiple myeloma, osteosarcoma, hemangioma, osteoid osteoma, aneurysmal bone cyst, chondrosarcoma, etc. [62].

Tumors of the spine most often initially present with a slow, gradual onset of back pain that is persistent at night and at rest [18]. Aside from pain, these can cause mechanical instability, vertebral fracture, and neurologic deficits if structural decomposition involves compression of the spinal cord or spinal nerves. In this case, the onset of pain may be abruptly acute and may involve radicular signs [63]. Studies have shown an association with poor quality of life (QoL) and functional status [64]. In patients with suspected spinal tumors, plain radiographs are first line, and usually help identify up to 80% of benign tumors and some malignancies [65]. Bone scintigraphy is helpful to identify sites of metastasis and primary origins. CT scan is the most advantageous to examine bone detail and mineralization; however, MRI is superior, especially in the evaluation of bone marrow, spinal canal, and the relationship of the tumor with adjacent structures and tumor vascularity [65]. Standardized assessments such as the Spine Instability Neoplastic score seek to provide reproducible estimates of metastatic vertebral instability to guide the need for immediate surgical fixation versus more conservation treatment options [66].

In settings of metastatic spine tumors, spinal surgery aims to correct spinal instability, decompress spaces, remove tumor growth, improve neurological function, and reduce pain [65]. It is crucial to recognize the poor functional status and limited life expectancy associated with vertebral metastases. Conventional surgery in this population is associated with prolonged recovery and significant complication rates and is therefore typically reserved for patients with neurological compromise and spinal instability [18,56,67]. Non-surgical therapies and treatments typically involve analgesics and bisphosphonates. External-beam radiation therapy is often used with variable results [64,68,69].

### 4.2. Spinal Tumor Ablation

**Figure 3:**
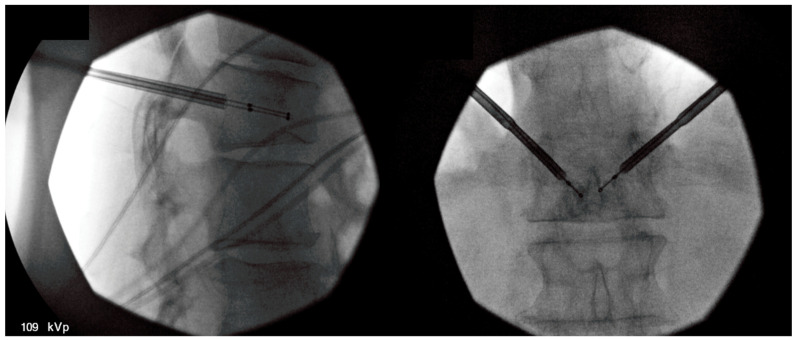
Fluoroscopic-guided vertebral body tumor ablation. The picture on the left shows a lateral view of bipedicular approach access, while the picture on the right shows an anterior–posterior (AP) view of the procedure with midline probe placement.

Spinal tumor ablation (STA) is an innovative, minimally invasive option to address pain from vertebral body tumors. Percutaneous treatment of non-spinal bone tumors was described first in 1992 with subsequent analysis of radiofrequency ablation (RFA) in a variety of non-spinal osseous structures [70,71]. The feasibility of STA was introduced in 2000 by Dupuy and colleagues with two human cases following an investigation on porcine models [72]. STA utilizes a percutaneous approach whereby one or more electrodes are inserted into an affected vertebra, and high-frequency alternating current ablates the tumor site. (Figure 3). Conventional radiofrequency causes coagulative necrosis with tissue temperatures of 50–100 °C [12,73,74,75]. Cryoablation applies a reverse technique for cell lysis with tissue temperatures reduced to −40 °C [76]. Subsequent pain relief is thought to derive from the destruction of periosteal nociceptors neural tissues involved in pain transmission. Either approach uses only a single outpatient treatment with mild to moderate sedation and local anesthetic. Several companies have created systems with radiofrequency, microwave, or cryoablation approaches. [13,72,74]. Treatment goals of STA may be a reduction in large tumor burden or as a definitive treatment for benign small tumors, such as osteoid osteomas or osteoblastomas [74]. Patient selection should include a comprehensive, interdisciplinary assessment of patient risk factors, medical comorbidities, and tumor burden. Generally accepted contraindications are active infection, coagulopathy, and contraindications to anesthesia or analgesia [77].

Most studies on STA are retrospective analyses. A few studies are worthy of a more detailed discussion. Anchala et al. published the first available multicenter retrospective analysis of STA with the majority (95%) of lesions also treated with augmentation [12]. The patient-reported visual analog scale (VAS) was significantly improved at 1 week and 1 and 6 months following the procedure. In sub-group analysis, 54% of patients decreased their use of analgesics. As augmentation was only performed in 95% of lesions, there was a note of two post-procedural vertebral fractures in cases where augmentation was not used.

Although radiofrequency alone has been demonstrated to decrease tumor size, ameliorate pain, and improve function, augmentation is often used during the same procedure. Cement (i.e., poly-methyl methacrylate) is typically chosen for its resistance to vertebral compressive forces, especially when addressing osteolytic metastases. There are limited studies with a head-to-head comparison of VA alone versus STA combined with augmentation, although the combination therapy supports enhanced pain reduction and either similar or improved benefit to functional and quality of life statuses [10,11,78].

It is important to note that osteoblastic metastatic lesions cause thickened bone that is resistant to the high-frequency alternating current applied in RFA. Therefore, cryoablation has been proposed as an alternative treatment approach. Tomaisian et al. used liquid argon to induce lesion temperature reduction via the cryoprobe tip during a series of freeze and thaw cycles [76]. This is thought to cause a transcellular osmotic gradient, membrane instability, and subsequent necrosis. This approach in 31 tumors throughout the spine resulted in significant decreases in numeric rating scores at 1 week, 1 month, and 3 months and persistent local tumor control in 30 cases after 10 months. A recent systematic review reported that microwave ablation technology might provide a possible advantage over other methods with larger ablation zones, shorter procedure times and potentially more effective ablative lesions with higher bony tissue impedance [79]. Adverse events in reported studies are rare, with the most common related to transient neuropathy or nerve injuries [10,13,14,16]. Dermal burns at the grounding pad site were noted in rare instances [16,68,71]. Limitations to many published studies are inherent to the severe underlying disease process, which is often fatal. Study populations are small, with limited follow-up periods and study drop-out related to deaths [20]. The diversity of primary tumors leads to heterogeneous study groups that often do not control for patient comorbidities, biological age, duration of malignancy diagnosis, or specific oncology treatments such as radiation therapy, corticosteroid use, or chemotherapy regimens. Furthermore, no randomized controlled trials met the inclusion criteria for this analysis. This should not negate the emphasis of rapid and sustained palliation of pain symptoms and improvement in function noted through multiple studies. Additionally, these treatment processes are inherently advantageous for feasible application under limited anesthesia or conscious sedation. Although no available studies randomize patients to surgical or percutaneous treatment, STA may offer benefits when in settings of poor surgical candidates.

### 4.3. Vertebral Fractures

Vertebral fractures (VFs) are among the leading causes of debilitating acute back pain in the elderly population. VFs are associated with limited function and poor quality of life and are prone to increased mortality over time [80,81,82,83,84]. Trends in VFs follow bone mineral density in general, affecting more women than men, especially in Caucasian and Asian populations, with increasing prevalence over 65 years of age [85]. VFs affect an estimated 1.5 million Americans annually [86].

**Figure 4:**
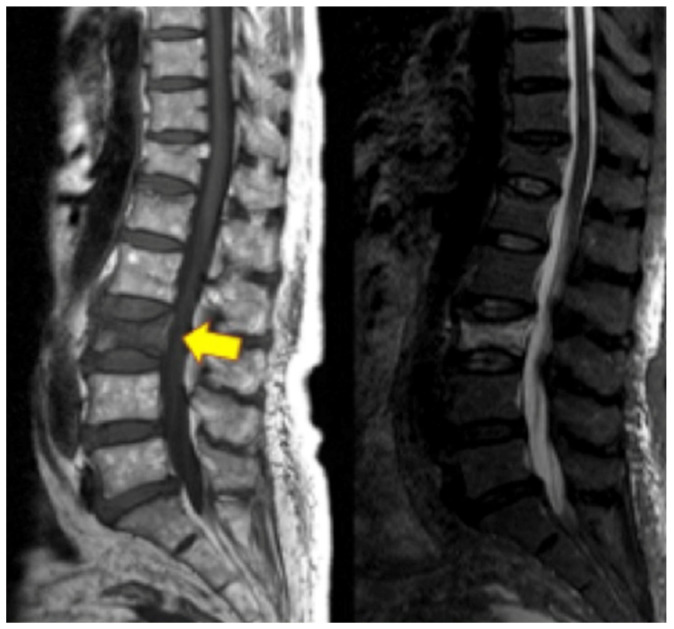
Lumbar spine MRI on the left with yellow arrowing pointing at the L3 vertebral compression fracture. On the right is an STIR image of the L3 vertebral compression fracture with hyperdense bone marrow changes representing acute vertebral compression fracture.

VFs may result from low-energy or high-energy trauma. Low-energy fractures are defined as fragility fractures, associated with decreased bone mineral density, infections, and cancer, while high-energy trauma is usually associated with high-impact axial loading with or without flexion, extension, or rotational components [87,88]. The most common etiology of VFs is osteoporosis; however, other etiologies include direct trauma, cancer, infection, steroids, chemotherapy or radiation, and other metabolic dysregulations [89]. Patients with VFs typically present with acute or chronic back pain, aggravated by prolonged standing, walking, or recurrent movements, and alleviated by rest and lying down. Additional symptoms depend on the spinal level of VF and whether it involves the anterior, middle, or posterior columns and the spinal canal, which, in this case, may include neurological findings. VFs may present with visible kyphotic deformity and increased pain with spinal percussion during physical exams [90]. At a minimum, thoracolumbar spine radiographs, including lateral, anterior–posterior, flexion, and extension, should be ordered if there is suspicion for VF. Additional diagnostic modalities include CT and/or MRI of the area to assess further bony detail, bone marrow edema, vertebral body height loss, etc. (Figure 4). These modalities are also more sensitive for early onset fracture compared to radiographs [91].

**Figure 5:**
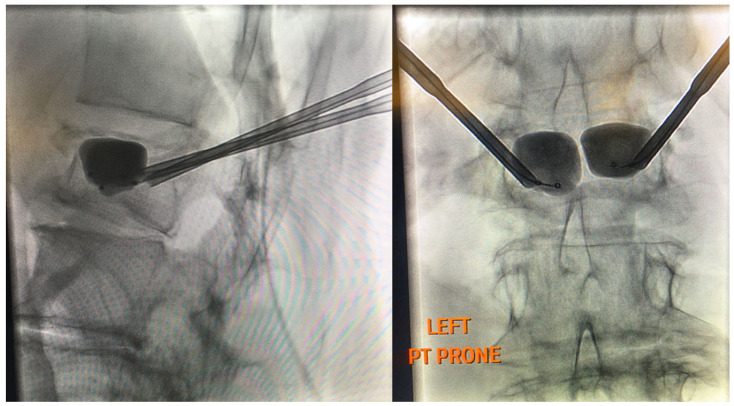
The image on the left is a lateral fluoroscopic view of the percutaneous balloon-kyphoplasty vertebral augmentation procedure. The image on the right is an anterior–posterior fluoroscopic view of the procedure demonstrating a bipedicular approach with vertebral height restoration.

Management of VF aims to reduce pain and the severe disability caused by the injury, improve range of motion and function, and restore quality of life to pre-injury level. Conservative treatment includes oral medications such as analgesics, gabapentinoids, hormone therapy with calcitonin, bisphosphonates, physical modalities, and bracing. Although the majority of VF may be managed with conservative non-surgical treatment, a subset of these with significant vertebral height loss, mechanical disruption, and uncontrollable pain (around 40%) may warrant minimally invasive vertebral augmentation (VA) (Figure 5) [92]. Despite the initial higher costs associated with interventional pain management, the overall expenditure associated with conservative care over a 4-year span and VA is similar [93]. Aside from socioeconomic costs, the QoL limitations associated with vertebral fractures must be weighed in the decision to optimize patient treatment [94,95]. Furthermore, although not fatal when in isolation, VFs are associated with increased mortality over time. Common causes of mortality in VFs include deep vein thrombosis or pulmonary embolism, and early treatment of VFs with VA has been shown to reduce mortality [84,96,97].

### 4.4. Vertebral Augmentation

VA has been evaluated extensively over the past two decades. The landmark VERTOS study compared percutaneous vertebroplasty (PVP) with optimal pain medication [41]. Immediate pain relief and improved mobility and function were seen with PVP compared to medication. short-duration trials also demonstrated superior pain reduction with VA compared to conservative care [37]. Klazen et al. (VERTOS II) and Blasco et al. also found sustained long-term benefit for QoL and pain scores in a combined 327 patients treated with PVP [33,36]. Subsequent studies extended the evaluation period, such as the 2009 FREE study [40]. VA compared to non-surgical management over 12 months revealed improved pain, mobility, QoL, and function, with no difference in adverse events ratio or frequency. Multiple other studies supported similar findings at 12 months [24,25,28,29,31,32]. Boonen et al. and Farrokhi et al. showed improved pain and functional scores in a combined 337 patients at all intervals over 24 and 36 months, respectively [34,35].

In 2016, Wang et al. compared VA with facet joint blocks [30]. Despite earlier pain relief with VA, there were no significant differences at long-term follow-up, suggesting that facet blocks may be a reasonable approach to address vertebral pain from VF when VA is contraindicated. However, facet blocks do not resolve important factors in VF related to pain, mobility, function, and QoL, such as correction of kyphosis, vertebral height restoration, kyphotic angle correction, and normalization of mechanical load. Therefore, VA remains the preferred intervention.

There are few studies that did not present favorable outcomes of VA compared to conservative treatment. Buchbinder et al. and Kallmes et al. reported no benefit with VA compared to sham treatment at short-term and long-term follow-ups, with similar improvements in pain and function in both groups [38,39]. The VERTOS IV RCT in 2018 compared 180 patients who underwent VA or sham and found no statistically significant decrease in pain or QoL scores at 12-month follow-up [26].

A substantial body of evidence favors the use of VA in the management of VF for clinical improvement. Overall mortality and health care cost optimization must also be considered [24,25,26,27,28,29,30,31,32,33,34,35,36,37,38,39,40,41,98,99,100,101,102,103]. Edidin et al. reported a 2.3–7.3-year life expectancy increase per patient in VA compared to conservative care [104,105,106]. Ong et al. noted over 50% reduction in 1-year mortality with VA compared to non-surgical management [84]. Cazzato et al. showed a 19% all-cause mortality risk reduction (RR) 36% morbidity decrease over 12 months in pooled data from 16 studies [107]. Hinde et al. determined a 22% reduction in mortality at 10 years after VA treatment [108]. Subgroup analysis also showed mortality benefits across 2- and 5-year periods.

Hopkins et al. compared VA to non-surgical treatment from a cost perspective in 7541 patients [109]. This demonstrated a higher short-term cost for VA. However, overall survival and quality-adjusted cost benefits of VA reduced expenditures over time compared to conservative treatment. Svedbom et al. studied data from the FREE and VERTOS II trials to arrive at a similar consensus [110]. Hirsch et al. concluded the number needed to treat (NNT) at 1 and 5 years was 14.8 and 11.9, respectively, to preserve one life with VA [96]. Overall, this emphasizes how VF can improve survival and decrease health care utilization.

### 4.5. Vertebrogenic Pain

**Figure 6:**
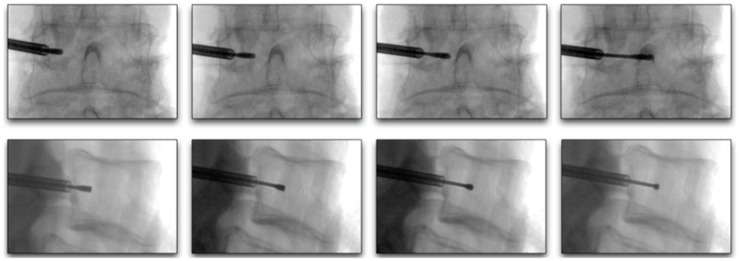
Lateral and AP fluoroscopy views of curved stylet advancement towards the ideal location between the 25–40% midline, between the anterior and posterior vertebral walls.

**Figure 7:**
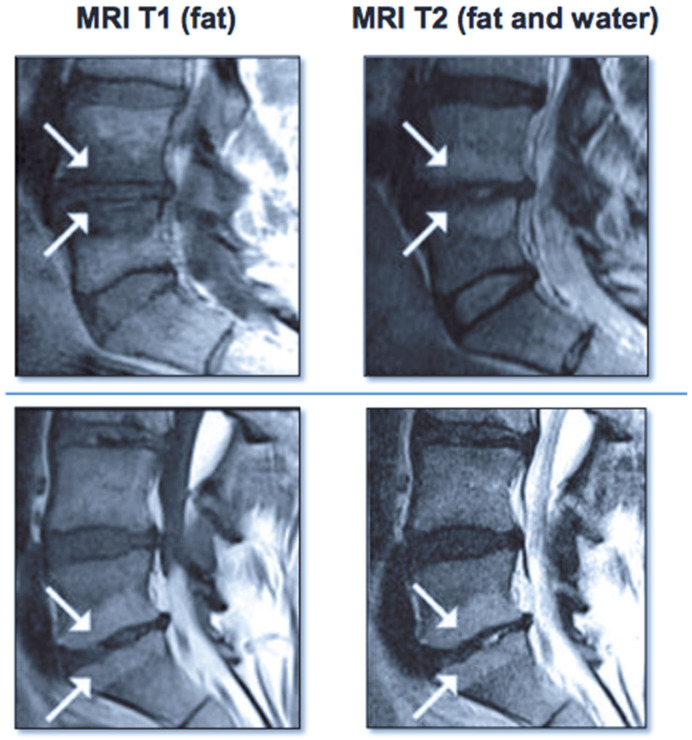
Images on the left show Modic type I changes in MRI with hypo-intense T1 signal Images on the right show Modic type II changes with hyper-intense T1 and T2 signals.

Vertebrogenic pain from endplate disruption is an etiology of chronic LBP that presents clinically different from other sources. Historically, the etiology of axial lumbar spine pain has been attributed to many anatomical structures, such as intervertebral disc degeneration, spinal canal narrowing, zygapophyseal joint pain, spinal ligaments hypertrophy, muscles and nerve root inflammation, etc. However, due to limited success with interventions targeting these structures, a recent shift in the vertebral pain treatment paradigm towards vertebral endplates has emerged. The basivertebral nerve (BVN) carries nociceptive input from damaged vertebral endplates related to inflammatory cytokines, substance *p*, and calcitonin gene-related peptide (CGRP), histologically confirmed with protein gene product (PGP) 9.5 positive staining under microscopy [111,112]. The BVN is a branch of the sinuvertebral nerve that enters the vertebral body and travels posterior-to-anterior to a bifurcation point about 50% into the vertebral body and divides cranially and caudally towards the endplates [113,114]. Basivertebral nerve ablation (BVNA) is a minimally invasive surgical treatment of vertebral pain performed similarly to vertebral augmentation and lumbar radiofrequency ablation, in the sense that it uses a transpedicular approach to the BVN bifurcation and delivers a high-frequency ablative lesion to interrupt nociceptive signaling from injured vertebral endplates (Figure 6) [115,116]. Vertebral endplates are highly vascularized structures that are particularly susceptible to post-traumatic degeneration, fissuring, intraosseous edema, and inflammatory changes [111,112,117,118,119]. These vertebral endplate changes have a specific phenotypic marker on MRI that directly correlates to vertebrogenic pain, known as Modic changes (MCs) type 1, type 2, and type 3 (Figure 7). Type 1 MCs manifest as the decreased signal intensity of fibrovascular intraosseous bone marrow edema on T1-weighted MRI sequences and as hyperintense or increased signal intensity on T2-weighted MRI sequences. Type 2 MCs represent fatty bone marrow infiltration and typically show an increased signal intensity in both T1 and T2 MRI sequence images in contrast to type 3 MCs that have decreased intensity in both MRI sequences [120,121,122]. Although MCs are radiological findings, their presence has been reported in up to 43% of subjects with spinal pain and is highly associated with this subset etiology [118,119,123,124]. Vertebrogenic pain from endplate damage presents clinically different than other etiologies of chronic LBP with reported painful episodes of greater duration and frequency and with significant functional impairment and disability compared to other etiologies. Pain tends to be axial and progressive in nature, aggravated by sitting, standing and spinal flexion and without radicular symptoms, numbness, tingling or motor weakness. This subset population tends to respond poorly to conservative treatment, epidural steroid injections, facet joint blocks and spinal surgery [112,118,119,121,123,124,125,126,127,128,129,130,131].

### 4.6. Basivertebral Nerve Ablation

Numerous clinical studies, reviews, meta-analyses, and society guidelines have reported the safety and clinical efficacy of BVNA in the treatment of vertebral pain [42,43,44,45,46,47,48,49,50,51,52,53,115,116,132,133,134,135,136]. Becker et al.’s study in 2017 reported that BVNA improved function at 6 weeks, 3, 6, and 12 months, with at least a 10-point reduction in ODI in 81% of subjects, as well as clinically meaningful improvement in pain scores and QoL [53]. The SMART study by Fischgrund et al. compared BVNA with sham treatment in a double-blind, prospective, randomized method [52]. BVNA treatment reduced ODI by 20 points, and up to 75% of subjects demonstrated a minimal clinically important improvement in pain at 6-month follow-up. However, SF-36 components were not statistically significant between the two arms [52]. Kim et al. noted statistically significant improvement in postoperative VAS, and 92.9% of subjects noted good to excellent outcomes by MacNab criteria following BNA [51]. Similarly, Truumees et al. noted significant improvement in pain QoL outcomes at 3-month follow-up [50]. Additionally, ODI was reduced by more than 10 points in 93% of subjects, and 75% reported greater than 20 points reductions. 50% of subjects discontinued opioid use after BVNA at the 3-month follow-up. Fischgrund et al. also reported similar continuous results with outcome changes at 24-month follow-up after BVNA with a mean 3.6 VAS reduction, 11.84 SF-36 average improvement, 46.4% opioid discontinuation, 60.7% opioid reduction, and 53.7% ODI mean reduction [49]. Similar results were seen in the INTRACEPT trial by Khalil et al. [48]. However, this study compared BVNA with the standard of care, including medications, therapy, manipulation, acupuncture and spinal injections. In the BVNA treatment arm, 62.7% of subjects reported greater than a 20-point reduction in contrast to 13.5% in the control group. In contrast to Truumees et al. and Fischgrund et al. (2019), this study found no significant difference in opioid reduction.

Several recent clinical studies, reviews, meta-analyses, and society guidelines reported BVNA safety and efficacy alone or in comparison to the standard of care for BVNA. Markman et al. reported a significant association between opioid utilization reduction and improved ODI post-procedure through a post hoc analysis of the Fischgrund et al. (2018) study [47]. Fischgrund et al. reported longer follow-up data in 2020, which allowed for analysis of health care utilization reduction following BVNA [45]. In the earlier study, 70% of subjects had chronic LBP despite spinal injections. The subsequent data showed only 4% of subjects received spinal injections after BVNA, suggesting that this intervention effectively reduces symptoms and minimizes additional health care costs. Smuck et al. reported BVNA superiority to the standard of care (medications, therapy, and spinal injections) at 3-, 6-, and 12-month intervals for improved pain, function and QoL [42]. However, opioid use did not differ between groups. Overall, BVNA is an effective intervention for the reduction of pain, disability, and improvement in function and QoL in a subset of patients with vertebral pain.

## 5. Limitations

This study is a scoping review that followed the PRISMA-ScR methodology. It is prudent to comment on the limitations of generalizability in such settings, as three different interventions were evaluated for respectively distinct vertebral pathologies. Therefore, a high level of heterogeneity is introduced, restricting further statistical analysis. A meta-analysis was not possible given the lack of standardization between studies, lack of control in some studies, and different patient selection criteria, treatment groups and outcome measurements.

## 6. Conclusions

The determination of the specific etiology of spinal pain remains a challenge despite its significant prevalence. The subset of these diagnoses attributed to vertebral etiologies from fractures, tumors, metastases, or vertebral endplate injury may be addressed with interventional options, including STA, VA, and BVNA. STA has the potential to reduce health care utilization while significantly improving immediate and sustained outcomes, including a reduction in opioid use, increased function, and improvement mood and QoL metrics. VA may reduce more than USD 1 billion spent annually addressing VF, as evidenced by multiple studies favoring its early use to reduce pain, improve QoL, facilitate ambulatory status and early mobilization, and ultimately improve morbidity and mortality in patients with VF. Finally, BVNA offers reproducible, sustainable, and clinically meaningful improvement in pain and function, with a few studies reporting reduced opioid consumption and disability and improvement in QoL.

## Figures and Tables

**Table 1:** Summary of findings: spinal tumor ablation.

Source, Year	Design	Sample Size	Treatment	Results	Adverse Events
Nakatsuka et al., 2009 [10]	Prospective CS	10 patients	Ablation alone in 4 patients Augmentation added in 6 patients	VAS: RFA: −2.6 (*p* = 0.0004) RFA + Augmentation: −4.9 (*p* = 0.003)	1 transient nerve injury
Proschek et al., 2009 [11]	Prospective CS	16 patients	Ablation alone in 8 patients Augmentation added in 8 patients	VAS: RFA: −3.9 (*p* = 0.008) RFA + Augmentation: −4.1 (*p* = 0.005) QoL Oswestry Index: RFA: improved 31% (*p* = 0.06) RFA + Augmentation: improved 31% (*p* = 0.071) All patients: reduction of pain (*p* = 0.0065) and an improvement in quality of life with less interference with daily activities (*p* = 0.043).	
Anchala et al., 2014 [12]	Retrospective CS	92 patients, 128 tumors	Ablation Augmentation added in 121 (95%) of lesions	VAS: −5.26 at 1 month (*p* < 0.001), only 83 patients included Analgesics: 54% patients decreased use 30% patients no change in use 16% patients increased use	2 vertebral fractures (both did not have augmentation)
Hillen et al., 2014 [13]	Retrospective CS	26 patients, 47 tumors	Ablation	VAS 7.82: −4.52 (*p* < 0.001) at 1 month 50% of patients decreased use of analgesics (27% increased, 23% unchanged)	4 transient radiculitis (all with intentional pedicular ablation)
Wallace et al., 2015 [14]	Retrospective CS	72 patients, 110 tumors	Ablation Augmentation added in 104 (95%) of cases	58 patients survived until 4-week follow-up NRS: −5.1 at 4 weeks (*p* < 0.0001) 45% (26/58) with complete relief Analgesic Use: Decreased in 31% (18/58) Increased activity: Increased in 50% (29/58)	4 transient radiculitis (pedicular ablations) 3/5 vertebrae not treated with immediate vertebral augmentation had fractures within 12 months
Bagla et al., 2016 [15]	Prospective CS	50 patients 69 tumors	Ablation Augmentation in 96% of vertebrae	NRS: −3.8 at 3 months (*p* < 0.0001) MODI: −15.9 at 3 months (*p* < 0.01) FACT-BP: +16.3 at 3 months (*p* < 0.0001)	1 post-STA pain related to an adjacent herniated disk (herniated prior to STA) 1 syncope
Khan et al., 2018 [16]	Retrospective CS	69 patients 102 tumors	Ablation + Augmentation	VAS: −5 ± 2.0 at 3–6 months MODI: −22 ± 12.8 at 3–6 months	1 S1 nerve thermal injury 1 skin burn
Reyes et al., 2018 [17]	Retropsective CS	49 patients 72 tumors	Ablation + Augmentation	VAS: −4.6 ± 3.4 (95% CI 3.6–5.6, *p* < 0.0001) ODI: −13.4 ± 8.1 (95% CI 10.4–16.4, *p* < 0.0001), only 30 patients included	NR
Tomasian et al., 2018 [18]	Retrospective CS	27 patients 33 tumors	Ablation + Augmentation	Radiographic tumor control: 96% (25/26) at 16 weeks	NR
Sayed et al., 2019 [19]	Prospective CS	30 patients, 34 tumors	Ablation Augmentation in 32/34 lesions	NRS-11: −3.16 (*p* < 0.01) at 3 months FACT-G7: +2.11 (*p* = 0.07) at 3 months	NR
Levy et al., 2020 [20]	Prospective CS	100 patients 134 tumors	Ablation Augmentation in 97% of cases (130/134)	3-month f/u in 42 vertebral patients BPI worst pain: −4.1 (95% CI 3.1–5.2, *p* < 0.0001) BPI average pain: −3.1 (95% CI 2.1–4.4, *p* < 0.0001) EQ-5D index +0.21 (*p* ≤ 0.003)	4 cases pneumonia/respiratory failure
Mayer et al., 2021 [21]	Retrospective CS	31 patients 37 metastases	Ablation + Augmentation	VAS: Clinical success of ≥3 VAS reduction in 80% on mean follow-up, 3.4 ± 2.9 months Prevention of tumor complications: 6/10 without residual or recurrent metastases at 3.8 ± 4.8 months Local tumor control for oligometastatic/oligoprogressive disease: 6/6 successful at of 5.0 ± 4.6 months	1 lethal sepsis from paravertebral abscess misdiagnosed day of procedure
Cazzato et a.l, 2021 [22]	Retrospective CS	23 patients 23 tumors	Ablation in 9 tumors Augmentation in 14 tumors	NRS: −3 at 31 ± 21 months (*p* < 0.001) Local progression: 3/7 (43%) tumors with curative ablation showed local progression at mean 4 ± 4-month follow-up 3/5 showed progression with RFA alone	1 post-operative pain condition 4 grade 2 peripheral neuropathies
Wu et al., 2021 [23]	Retrospective CS	23 patients 33 tumors	Ablation + Augmentation	VAS: −5.7 at 24 weeks (*p* < 0.001) Daily morphine dose: −91.3 at 24 weeks (*p* < 0.001) ODI: −25.1 at 24 weeks (*p* < 0.05)	1 skin infection at puncture site

Legend: CS—Comparative study VAS—Visual Analog Scale; NRS—Numeric Rating Scale; ODI—Oswestry Disability Index MODI—Modified Oswestry Disability Index BPI—Brief Pain Inventory; NR—None Reported; FACT-G7—Functional Assessment of Cancer Therapy-General 7 Item Version; FACT-BP—Functional Assessment of Cancer Therapy-Bone Pain.

**Table 2:** Summary of findings: vertebral augmentation.

Source, Year	Design	Sample Size	Treatment Arms	Results	Adverse Events
Beall et al., 2019 [24]	MC PR	350	PBK	Statistically significant improvement at 3 months: NRS—improved 6 points (*p* < 0.001) ODI—improved 35.3 points (*p* < 0.001) SF-36v2 PCS—improved 12.4 points (*p* < 0.001) EQ-5D—improved 0.351 points (*p* < 0.001) Statistically significant improvement noted at all time points	1 asymptomatic balloon rupture 1 subject with rib pain beginning intraoperatively ending < 6 months 1 Adjacent VF 1 aspiration pneumonia with prolonged hospital stay 1 myocardial infarction at 105 days postop
Liu et al., 2019 [25]	RCT	116	PBK vs. CT	VAS (after treatment) Observation: 2.25 ± 0.21 Control: 4.54 ± 0.28 Trailing Edge (%) Observation: 10.14 ± 3.19 Control: 1.84 ± 0.67Leading Edge (%) Observation: 15.13 ± 4.21 Control: 0.74 ± 0.47 Midcourt Line Height (%) Observation: 14.72 ± 3.25 Control:1.73 ± 0.53 Upper Thoracic Kyphosis (°) Observation: 13.17 ± 2.67 Control:1.69 ± 0.83 Barthel Index Observation: 24.34 ± 4.53 Control: 31.57 ± 4.25	Observation Group: 1 cement leakage. Rate of complication 1.72% Control Group: 1 venous embolism, 4 decubitus ulcers and 4 infections Rate of complication 15.52% Observation had significantly lower rates of complications (*p* < 0.05)
Firanescu et al., 2018 [26]	RCT DB	180	PVP vs. Sham	Mean QUALEFFO reduction at 12 months: PVP: 18.32 (95% CI 18.32 to 23.61) Sham: 18.61 (95% CI 13.02 to 24.2) Difference: −0.14 (95% CI −3.04 to 2.76) Mean RMDQ reduction at 12 months: PVP: 7.71 (95% CI 5.87 to 9.55) Sham: 7.47 (95% CI 5.56 to 9.38) Difference: 0.12 (95% CI −1.11 to 1.35) Mean VAS reduction at 12 months): PVP: 5.00 (95% CI 4.31–5.70) Sham: 4.75 (95% CI 3.93–5.57) Difference: 0.13(95% CI −0.41 to 0.66)	1 patient with COPD developed respiratory insufficiency 1 patient had a vasovagal reaction
Hansen et al., 2016 [27]	RCT DB	46	PVP vs. Sham	Mean SF-36 PCS (SE) at 12 months): PVP: 31.90 (9.19) Sham: 35.15 (11.92) No statistical difference between groups Mean SF-36 MCS (SE) at 12 months: PVP: 48.60 (10.75) Sham: 53.60 (10.29) No statistical difference between groups Mean EQ-5D (SE) at 12 months: PVP: 0.67 (0.27) Sham: 0.74 (0.22) No statistical difference between groups Mean VAS (SE) at 12 months: PVP: 28.35 (5.16) Sham: 30.67 (4.65) Statistical difference between groups	NR
Clark et al., 2016 [28]	RCT MC DB	120	PVP vs. Sham	RMDQ: Mean reduction greater in vertebroplasty group. Maximum difference at 6 months of 4.2 (95% CI 1:6 to 6:9, *p = 0*.0022) QUALEFFO: Lower in vertebroplasty group with mean difference at 6 months of 7 (95% CI 1–13, *p* = 0.032) EQ-5D Higher score at 1 and 6 months (−0.06, 95% CI −0.10 to −0.01, *p* = 0.012) NRS: Mean reduction ratio for vertebroplasty to placebo 1.3 (95% CI 0—2.6, *p* = 0.043) VAS: Lower score with vertebroplasty at: 14 days (95% CI 6–39 *p* = 0.01) 6 months (11, 95% CI 0–23, *p* = 0.050)	3 patients in each group died from unrelated causes Vertebroplasty Group: 1 respiratory arrest after sedation (resuscitated and underwent procedure 2 days later) 1 supracondylar humerus fracture during Placebo Group: 2 cases of spinal cord compression from interval collapse and retropulsion
Leali et al., 2016 [29]	RCT MC PR	400	PVP vs. CT	Mean ODI: 31.7% (Post-Op), 53.6% (Pre-Op), *p* < 0.012 Mean VAS: 2.3 points (Post-Op), 4.8 (Pre-Op), *p* < 0.023 Analgesia: 120 (65%) able to stop analgesia after 48 h (*p* < 0.0001)	1 fracture of transverse process 1 psoas muscle bleed 3 VFs
Wang et al., 2016 [30]	RCT PR	206	PVP vs. Image-guided facet joint blocks	Statistically significantly lower VAS, ODI, and RMDQ in PVP group compared to FB group at 1 week (*p* < 0.05). No statistical significance between groups for VAS, ODI, SF-36 at 12 months (*p* > 0.05)	NR
Yang, et al., 2016 [31]	RCT PR	135	PVP vs. CT	Statistically significant improvement for VAS, ODI, and QUALEFFO at 12 months (*p* < 0.0001)	NR
Chen et al., 2014 [32]	RCT CS	96	PVP vs. CT	VAS, ODI, RMDQ significantly better at 12 months in PVP group (*p* < 0.001) 39 PVP patients experienced complete pain relief compared to 15 CT patients (*p* < 0.001	NR
Blasco et al., 2012 [33]	RCT PR	125	PVP vs. CT	QUALEFFO: PVP group had significant improvement compared to CT at 6 & 12 months VAS at 2 months: 42% mean reduction with PVP group compared to only 25% in CT group (*p* = 0.035) Analgesia: No significant difference between two groups New Fractures: 2.78-fold more risk of new fracture in PVP group	NR
Boonen et al., 2011 [34]	RCT	232	PBK vs. CT	SF-36: Significant improvement in pain (3.24 points, 95% CI 1.47–5.01, *p* = 0.0004) EQ-5D: Significant improvement in QoL (0.12 points, 95% CI 0.06–0.18, *p* = 0.0002) RMDQ: −3.01-point difference in reduction of disability (95% CI −4.14 to −1.89, *p* < 0.001) VAS: Significant reduction in back pain (−1.49 points, 95% CI −1.88 to −1.10, *p* < 0.0001) Likert Scale: Patients more satisfied (3.09 points, 95% CI 2.26–3.92, *p* < 0.0001)	Similar frequency of adverse events and serious adverse events between two groups 1 hematoma at surgical site 1 recurrent UTI within 2 days of surgery. This patient also developed spondylitis 23 deaths (12 in observation group and 11 in control group) that were all unrelated to treatment
Farrokhi et al., 2011 [35]	RCT CS	105	PVP vs. OPM	ODI Mean Difference: −14.0 (−14.91 to −13.09, *p* < 0.01) VAS Mean Difference: −1.5 (−9.85 to 6.85, *p* < 0.81) Vertebral Height Mean Difference (cm): 2.0 (1.5 to 0.44, *p* < 0.01) Sagittal Index Mean Difference:: −14.0 (−14.96 to −13.05, *p* < 0.011)	1 patient with epidural cement leakage
Klazen et al., 2010 [36]	RCT MC CS	202	PVP vs. CT	EQ-5D: 1 month—favored vertebroplasty with difference of 0.010 (95% CI 0.014–0.006) 1 year—favored vertebroplasty with difference of 0.108 (0.177–0.040) QUALEFFO and RMQD: Vertebroplasty had greater improvement (and quicker) over time VAS at 1 Month: Vertebroplasty—−5.2 (95% CI −5.88 to −4.72) Conservative—−2.7 (95% CI −3.22 to −1.98) Difference—2.6 (95% CI 1.74–3.37, *p* < 0.0001) VAS at 1 year: Vertebroplasty—−5.7(95% CI −6.22 to −4.98) Conservative—−3.7 (95% CI −4.35 to −3.05) Difference—2.0 (95% CI 1.13–2.80, *p* < 0.001)	NR
Rousing et al., 2010 [37]	RCT	50	PVP vs. CT	VAS: PVP: 1.8 (95% CI 0.8–2.8) CT: 2.6 (95% CI 1.2–4.0) *p* = 0.33	2 adjacent VFs
Buchbinder et al., 2009 [38]	RCT MC DB	71	PVP vs. Sham	QUALEFFO Score: PVP: 6.4 ± 13.4 Sham: 6.1 ± 13.4 Difference: 0.6 (95% CI −5.1 to 6.2) AQoL Score: PVP: 0.0 ± 0.3 Sham: 0.1 ± 0.3 Difference: 0.1 (95% CI −0.1 to 0.2) RMDQ Score: PVP: 4.1 ± 5.8 Sham: 3.7 ± 5.8 Difference: 0.0 (−3.0 to 2.9) EQ-5D Score: PVP: 0.2 ± 0.4 Sham: 0.2 ± 0.4 Difference: 0.0 (−0.1 to 0.2) Pain Score: PVP: 2.4 ± 3.3 Sham: 2.1 ± 3.3 Difference: 0.1 (95% CI −1.2 to 1.4)	7 VFs 3 new rib fractures 1 case of osteomyelitis
Kallmes et al., 2009 [39]	RCT MC	131	PVP vs. Sham	Pain Intensity: PVP: 3.9 ± 2.9 Sham: 4.6 ± 3.0 Treatment effect: 0.7 (−0.3 to 1.7, *p* = 0.19) RDQ: PVP: 12.0 ± 6.3 Sham: 13.0 ± 6.4 Treatment Effect: 0.7 (95% CI −1.3 to 2.8, *p* = 0.49)	1 thecal sac injury 1 patient admitted with tachycardia and rigors
Wardlaw et al., 2009 [40]	RCT CS	300	PBK vs. CT	SF-36 1 month: PBK: 7.2 (95% CI 5.7–8.8) CT: 2.0 (95% CI 0.4–3.6) *p* < 0.0001 SF-36 12 month: Difference 1.5 (95% CI −0.8–3.9) *p* = 0.208 VAS 12 months: PBK > CT decrease 0.9 (CI 95% 0.3–1.5) *p* = 0.0034	1 hematoma 1 UTI
Voormolen et al., 2007 [41]	RCT CS	34	PVP vs. OPM	QUALEFFO: PVP: −6.8 OPM: −0.7 Difference: −6.1 (95% CI −10.7 to −1.6) RMD: PVP: +19 OPM: −2 Difference: 21 (95% CI 0.07 to 0.35 VAS: PVP: −2.1 OPM: −1.1 Difference: −1.5 (95% CI −3.2 to 0.2) Analgesic Use: PVP: −0.7 OPM: +0.9 Difference: −1.5 (95% CI −2.3 to −0.8)	2 VFs

Legend: RCT—Randomized control trial; DB—Double blind; CS—Comparative study; MC—Multicenter; PR—Prospective; CT—Conservative treatment; OPM—Optimal Pain medication PVP—Percutaneous vertebroplasty; PBK—Balloon Kyphoplasty; VF—Vertebral fracture; NR—None Reported.

**Table 3:** Summary of findings: basivertebral nerve ablation.

Source, Year	Design	Sample Size	Treatment Arms	Results	Complications
Smuck et al., 2021 [42]	PR MC multicenter open label RCT	140	BVN ablation and standard care	Superiority of BVN ablation at 3 months for the primary endpoint Mean ODI reduction, difference between arms of −20.3 (CI −25.9 to −14.7 points; *p* < 0.001) Mean VAS pain improvement (difference of −2.5 cm between arms (CI −3.37 to −1.64, *p* < 0.001)	No serious adverse events
Macadaeg et al., 2020 [43]	PR open-label, single-arm, MC	47	Transpedicular Radiofrequency of Basivertebral Nerve	Mean ODI change of −32.6 Mean VAS change of −4.3 Responder Rates: 15-point ODI reduction—88.9% 20-point ODI reduction—88.4% 2.0 cm VAS reduction—80.0% SF-36 Total Score increase of 26.3 EQ-5D-5L increase of 0.22	No serious device-related or device-procedure-related adverse events
De Vivo et al., 2021 [44]	PR uncontrolled trial	56	Percutaneous Radiofrequency Ablation of Basivertebral Nerve	Mean ODI change of −32.4 Mean VAS change of −4.3 Responder Rates: 10-point ODI reduction—96.4% 2-point VAS reduction—96.4%	No serious adverse events. No abnormalities on 3-month CT. No bone weakening on density analysis.
Fischgrund et al., 2020 [45]	Open-label follow-up study of RCT treatment arm	100	Transpedicular Radiofrequency of Basivertebral Nerve	Mean ODI change of −25.9 Mean VAS change of −4.4 Responder Rates: 15-point ODI reduction—77% 2-point VAS reduction—88% Combined (ODI ≥ 15 and VAS ≥ 2)—75% In patients on opioids at baseline: Stopped use—73.3%	No serious device-related adverse events
Kim et al., 2020 [46]	PR case series	30	Transforaminal or Interlaminar Endoscopic Radiofrequency Ablation of Basivertebral and Sinuvertebral Nerves	Mean ODI change of −52.7 Mean VAS change of −5.7 McNab’s Criteria: Excellent outcomes—56.7% Good outcomes—36.7% Fair outcomes—6.7%	Not Reported
Markman et al., 2020 [47]	Post-hoc analysis of sham-controlled trial	69	Transpedicular Radiofrequency of Basivertebral Nerve vs. Sham Control	Treatment arm: Decreased opioid use (*n* = 27) mean ODI change −24.9 Increased opioid use (*n* = 18) mean ODI change −7.3 Sham arm: Decreased opioid use (*n* = 19) mean ODI change −17.4 Increased opioid use (*n* = 5) mean ODI change −1.2	Not Assessed
Khalil et al., 2019 [48]	PR MC randomized	104	Transpedicular Radiofrequency of Basivertebral Nerve vs. Standard Care Control	Mean ODI change of −25.3 (treatment) vs. −4.4 (control) Mean VAS change of −3.5 (treatment) vs. −1.0 (control)Responder Rates: 20-point ODI reduction—62.7% (treatment) vs. 13.5% (control) 2-point VAS reduction—72.5% (treatment) vs. 34.0% (control)SF-36: PCS—increase 14.02 (treatment) vs. 2.114 (control) MCS—increase 2.615 (treatment) vs. 2.786 *decrease* (control) EQ-5D-5L Increase 0.1803 (treatment) vs. 0.0135 (control) No change in opioid use in either arm at 3 months	No serious device-related or serious device-procedure-related adverse events
Fischgrund et al., 2019 [49]	Open-label follow-up study	106	Transpedicular Radiofrequency of Basivertebral Nerve	Mean ODI change of −23.4 Mean VAS change of −3.6 Responder Rates: 10-point ODI reduction—76.4% 20-point ODI reduction—57.5% 1.5 cm VAS reduction—70.2% SF-36 PCS increase of 11.84 In patients on opioids at baseline: Reduced use—60.7% Stopped use—46.4%	No device or procedure-related patient deaths, no unanticipated adverse device effects, and no device-related serious adverse events (SAEs).
Truumees et al., 2019 [50]	PR, MC, open-label, single-arm	28	Transpedicular Radiofrequency of Basivertebral Nerve	Mean ODI change of −30.1 Mean VAS change of −3.5 Responder Rates: 10-point ODI reduction—92.9% 20-point ODI reduction—75.0% 2.0 cm VAS reduction—75.0% SF-36 PCS increase of 15.78 SF-36 MCS increase of 4.23 EQ-5D-5L increase of 0.198 50% (4/8) patients taking extended-release narcotics had stopped by 3 months post procedure.	No serious device-related or device-procedure-related adverse events
Kim et al., 2018 [51]	Single-center, retrospective observational	14	Transforaminal Epiduroscopic Basivertebral Nerve Laser Ablation	Mean VAS change of −5.4 McNab’s Criteria: Excellent outcomes—50.0% Good outcomes—42.9% Fair outcomes—7.1%	There were no occurrences of infections, discitis, paresis, dural tears, vascular injuries, or systemic complications. There were no serious device or procedure-related adverse events.
Fischgrund et al., 2018 [52]	PR MC RCT, double-blind, sham-controlled	225	Transpedicular Radiofrequency of Basivertebral Nerve vs. Sham Control	3 Month Primary Endpoint (per protocol): Mean ODI change of −20.3 (treatment) vs. −15.4 (control) Mean VAS change of −2.9 (treatment) vs. −2.5 (control) 12 Month Primary Endpoint (per protocol): Mean ODI change of −19.8 (treatment) vs. −15.9 (control) Mean VAS change of −2.8 (treatment) vs. −2.2 (control)Responder Rates 3 Month (per protocol): 10-point ODI reduction—75.6% (treatment) vs. 55.3% (control) SF-36—3 Month (per protocol): PCS—increase 9.74 (treatment) vs. 9.05 (control) MCS—increase 2.24 (treatment) vs. 0.78 (control) SF-36—12 Month (per protocol): PCS—increase 9.17 (treatment) vs. 7.63 (control) MCS—increase 1.13 (treatment) vs. 1.46 *decrease* (control)	No serious device-related adverse events1 serious device procedure adverse event: 1—vertebral compression fracture (sham)
Becker et al., 2017 [53]	PR, MC, single-arm	16	Transpedicular and Extrapedicular Radiofrequency of Basivertebral Nerve	Mean ODI change of −29 Mean VAS change of −16 mm Responder Rates: 10-point ODI reduction—81% SF-36 PCS increase of 7.2	No access-related complications. No reports of thermal or non-thermal injuries. No compression fractures (per independent radiology lab)

Legend: RCT—Randomized control trial; MC—Multicenter; PR—Prospective ODI—Oswestry disability index; VAS—visual analog scale BVN—basivertebral nerve; SF-36—short form 35; MCS—mental health component; PCS—physical component; EQ-5D-5L—quality of life function questionnaire.
